# Supplementary material for: Volatiles and Antifungal-Antibacterial-Antiviral Activity of South African Salvia spp. Essential Oils Cultivated in Uniform Conditions
Source: Molecules. 2021 May 10;26(9):2826. doi: 10.3390/molecules26092826 (PMC8126244; doi:10.3390/molecules26092826)
Supplement: Supplementary file 1 [file molecules-26-02826-s001.zip › molecules-1180094-supplementary.pdf]

molecules

MDPI

Supplementary Information

Volatiles and Antifungal–Antibacterial–Antiviral Activity of South African *Salvia* spp. Essential Oils Cultivated in Uniform Conditions

Basma Najar [1,*], Giulia Mecacci [1], Valeria Nardi [1], Claudio Cervelli [2], Simona Nardoni [3], Francesca Mancianti [3,4], Valentina Virginia Ebani [3,4], Simone Giannecchini [5] and Luisa Pistelli [1,4]

[1] Department of Pharmacy, University of Pisa, Via Bonanno Pisano 33, 56126 Pisa, Italy; g.mecacci@studenti.unipi.it (G.M.); nardivaleria12@gmail.com (V.N.)

[2] Council for Agricultural Research and Economics (CREA), Corso Inglesi 508, 18038 Sanremo, Italy; claudio.cervelli@crea.gov.it

[3] Department of Veterinary Sciences, University of Pisa, Viale delle Piagge 2, 56124 Pisa, Italy; simona.nardoni@unipi.it (S.N.); francesca.mancianti@unipi.it (F.M.); valentina.virginia.ebani@unipi.it (V.V.E.)

* Correspondence: Research Center "Nutraceutical and Food for Health", University of Pisa, Via del Borghetto, 80, 56126 Pisa, Italy; luisa.pistelli@unipi.it

* Correspondence: of Experimental and Clinical Medicine, University of Florence, viale Morgagni 48, 50134 Florence, Italy; simone.giannecchini@unifi.it

* Correspondence: basma.najar@farm.unipi.it; Tel.: +39-334-809-3486

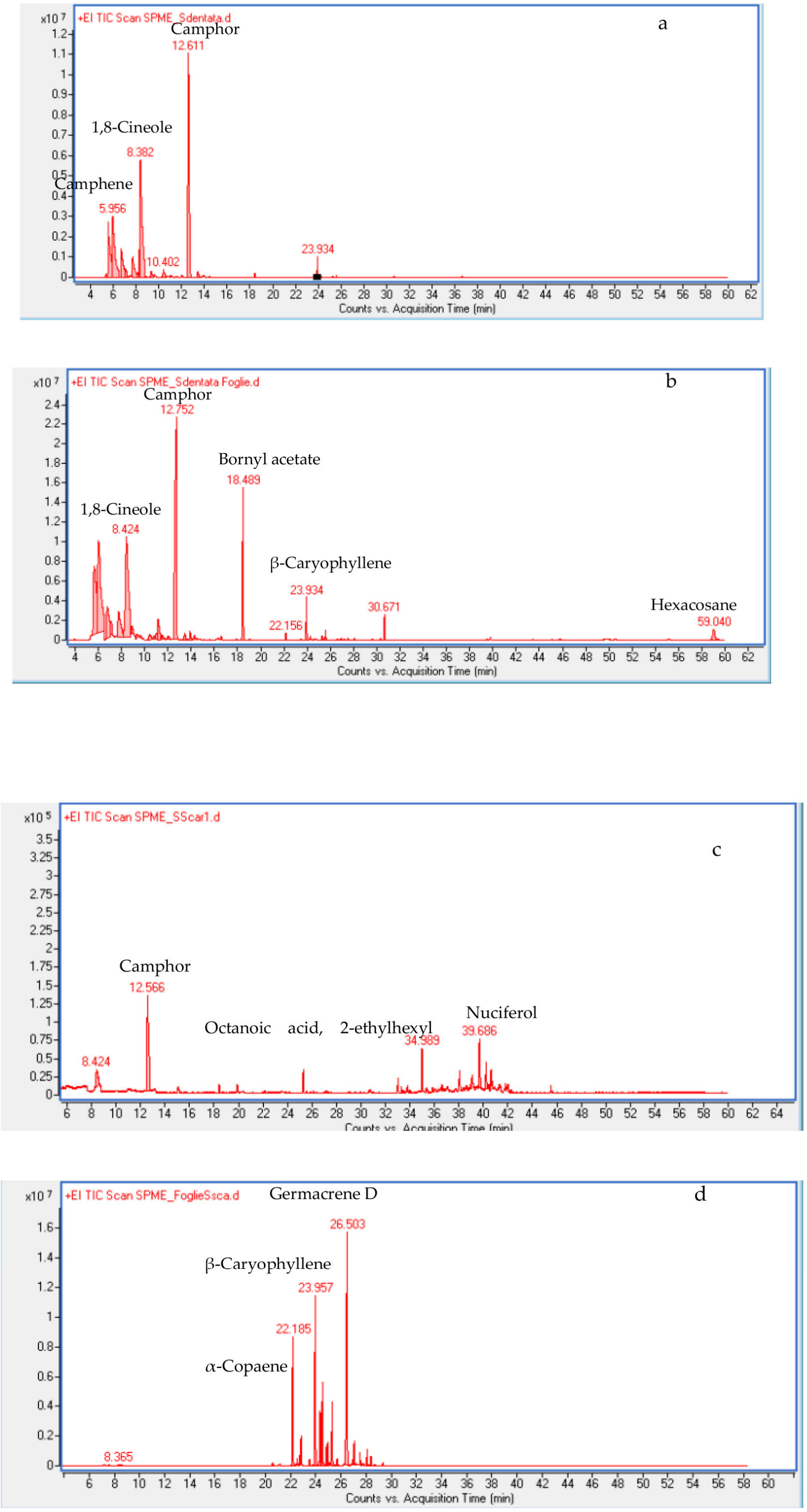

Figure S1. The aroma profile chromatograms of the studied sage. a: Flowers *S. dentata*, b: Leaves *S. dentata*, c: Flowers *S. scabra*, d. Leaves *S. scabra*.

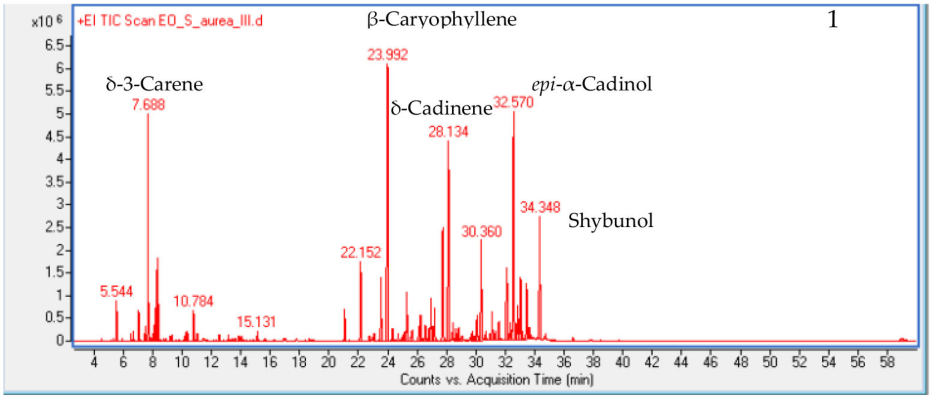

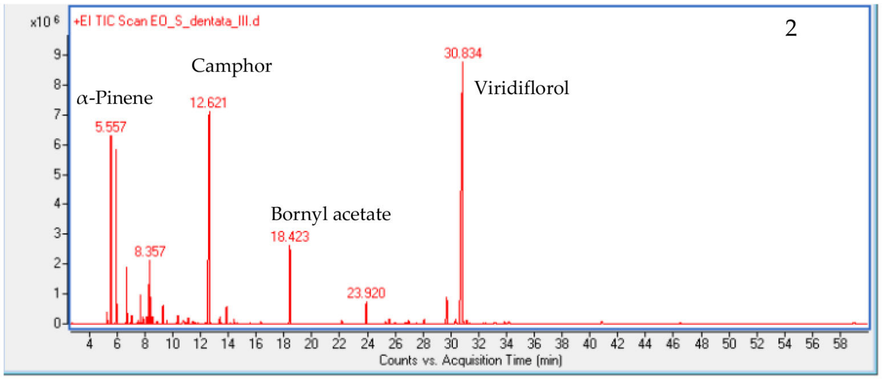

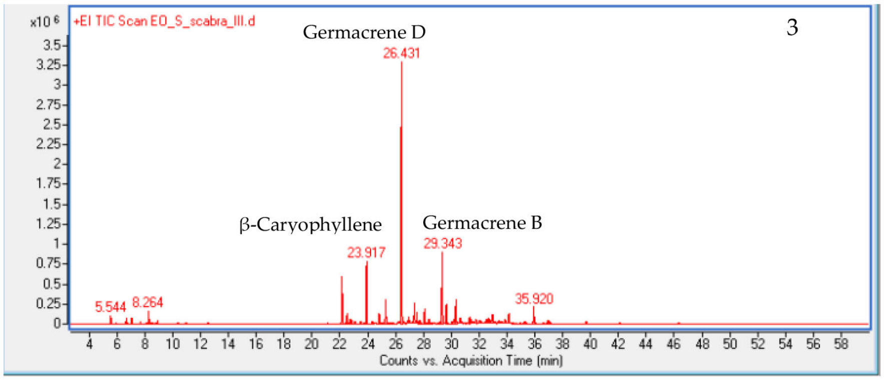

Figure S2. The essential oil chromatograms of the studied sage species. S1: *S. aurea*, S2: *S. dentata*, S3: *S. scabra*.

Table S1. Monographs of the studied *Salvia* species.

Sage species	Characteristics
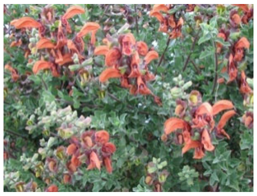 ***Salvia aurea* L. (= *Salvia africana-lutea* L.)**	**Voucher N°: HMGBH.e/7290.2019.001**<br>**Common name:** "bruinsalie", "sandsalie", "strandsalie" and "geelblomsalie" (www.sanbi.org, accessed 15 April 2021).<br>**Distribution:** typical of arid and rocky coastal environments, introduced in North America and Europe as an ornamental plant and has successfully adapted to the Mediterranean climate [26].<br>**Botanical description:** It is a very branched suffrutice, with an enlarged habit, and can reach 90 cm in height. The leaves vary in shape, from oval to rounded, with unevenly toothed margins, rich in coating and glandular hairs, which give them a silver color. The flowers, grouped in verticils, have large bell-shaped calyxes that tend to take on a papery appearance with aging. The corollas, pressed on top of each other, are yellow when the flower is in bud and become golden brown when ripe [66,67].<br>**Traditional uses:** used as an infusion for the treatment of colds, chronic bronchitis, tuberculosis, flu, fever, headache and digestive and gynecological disorders [68]. Promising activity against *Mycobacterium aureum* and *Mycobacterium tuberculosis* [69]. In-vivo tests demonstrate the efficacy of the aqueous extract as an analgesic and antipyretic [70].
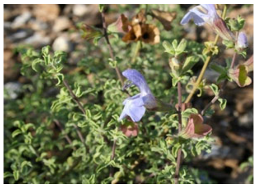 ***Salvia dentata* Aiton**	**Voucher N°: HMGBH.e/7290.2019.002**<br>**Common name:** “bergsalie” e “blousalie”<br>Distribution: It prefers the arid and rocky slopes of the Western Cape Province and is found at an altitude between 150 and 1500 m. It was first described in 1789 by the Scottish botanist William Aiton (1731-1793).<br>**Botanical description:** An evergreen shrub It is an upright plant and can reach 2 m in height.<br>The leaves and stems are grey-green due to the presence of glandular hairs, but become hairless when ripe. The stems are unusually rounded. The leaves are curled at the apex, narrow and irregular in shape; the margin is mainly toothed, a characteristic which gives it its name. The flowers have a green calyx or with violet hues and a purple or white corolla that reaches 25 cm in length; the upper lip is slightly curved downwards, while the lower lip is flattened to facilitate access to pollinating insects. The seeds, black or dark brown, are clearly visible at the base of the glass. Flowering occurs between July and January (www.sanbi.org, accessed 15 April 2021).<br>**Traditional uses:** the decoction of leaves is used as a tonic and for the treatment of colds, back pain and kidney problems; it is often associated with other herbs [71].

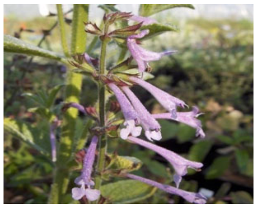<br>***Salvia scabra* Thunb.**	**Voucher N°: HMGBH.e/7290.2019.003**<br>**Common name:** "kuskruipsalie" e "usikiki"<br>**Distribution:** grows wild on the rocky slopes and sandy coasts of the Eastern Cape Province in South Africa. It prefers open and sunny places and is located at an altitude between 15 and 450 m.<br>**Botanical description:** is a perennial herbaceous plant, usually it does not exceed 50 cm in height. The stems, erect, woody and very branched, are covered with velvety and dense covering hairs. The leaves are green, rough, lyrate and lobed, with crenate or wavy margin. Both leaf surfaces are covered with coating hairs, but only on the underside are glandular hairs present. The flowers, gathered in verticillastri, have a tubular corolla, lilac, mauve or purple in color, with a deeply bidentate and very protruding lower lip (Sotti, 1997; [65]). The glass has hairs and red-violet shades. Flowering occurs between spring and autumn, with a peak during the summer. The plant produces a large amount of dark brown seeds (www.sanbi.org, accessed 15 April 2021).<br>**Traditional uses:** The leaves decoction is used as a purgative [20].

References

20. Kamatou, G.P.P.; Van Zyl, R.L.; Davids, H.; Van Heerden, F.R.; Lourens, A.C.U.; Viljoen, A.M. Antimalarial and anticancer activities of selected South African *Salvia* species and isolated compounds from *S. radula. South African J. Bot.* **2008**, *74*, 238–243, doi:10.1016/j.sajb.2007.08.001.
26. Serrato-Valenti, G.; Bisio, A.; Cornara, L.; Ciarallo, G. Structural and histochemical investigation of the glandular trichomes of *Salvia aurea* L. leaves, and chemical analysis of the essential oil. *Ann. Bot.* **1997**, *79*, 329–336, doi:10.1006/anbo.1996.0348.
66. Benvenuti E., *Mille Salvie.* Maestri di Giardino Associazione Culturale, EAN: 9788898150014, Cuneo, Italy, 2012.
67. Sotti, M. L. *Salvie,* Edagricole, Italy, 1997.
68. Arief, M.M.H.; Abdel, A.; Hussein, F.; Mohammed, A.; ElMwafy, H.M. Chemical and Bioactivity Studies on Salvia Africa-na-Lutea: Cytotoxicity and Apoptosis Induction by Abietane Diterpenes Isolated from Salvia Africana-Lutea. *J. Bas. & Environ. Sci.* **2018**, *5*, 72–79.
69. Seaman, T. The antimicrobial and antimycobacterial activity of plants used for the treatment of respiratory ailments in South-ernAfrica and the isolation of anacardic acid from Ozoroa paniculosa. M.Sc. Dissertation. University of the Witwatersrand, Johannesburg, South Africa, 2005.
70. Amabeoku, G.J.; Eagles, P.; Scott, G.; Mayeng, I.; Springfield, E. Analgesic and antipyretic effects of Dodonaea angustifolia and Salvia africana-lutea. *J. Ethnopharmacol.* **2001**, *75*, 117–124, doi:10.1016/s0378-8741(00)00395-0.
71. De Beer, J.J.J.; Van Wyk, B.-E. An ethnobotanical survey of the Agter–Hantam, Northern Cape Province, South Africa. *South Afr. J. Bot.* **2011**, *77*, 741–754, doi:10.1016/j.sajb.2011.03.013.
